# The SARS-CoV-2 envelope and membrane proteins modulate maturation and retention of the spike protein, allowing assembly of virus-like particles

**DOI:** 10.1074/jbc.RA120.016175

**Published:** 2020-12-03

**Authors:** Bertrand Boson, Vincent Legros, Bingjie Zhou, Eglantine Siret, Cyrille Mathieu, François-Loïc Cosset, Dimitri Lavillette, Solène Denolly

**Affiliations:** 1 CIRI – Centre International de Recherche en Infectiologie, Univ Lyon, Université Claude Bernard Lyon 1, Inserm, U1111, CNRS, UMR5308, ENS Lyon, Lyon, France; 2 Université de Lyon, VetAgro Sup, Marcy-l'Étoile, France; 3 Institut Pasteur of Shanghai, Chinese Academy of Sciences, University of Chinese Academy of Sciences, Shanghai, China; 4 CAS Key Laboratory of Molecular Virology & Immunology, Institut Pasteur of Shanghai Chinese Academy of Sciences, Pasteurien College, Soochow University, Jiangsu, China

**Keywords:** virus assembly, viral protein, glycoprotein, secretion, infectious disease, COVID-19, SARS-CoV, DMEM, Dulbecco’s modified minimal essential medium, E, envelope, ERGIC, endoplasmic reticulum (ER)–Golgi intermediate compartment, IBV, infectious bronchitis virus, IF, immunofluorescence, M, membrane, MERS-CoV, Middle East Respiratory Virus, N, nucleoprotein, SARS-CoV-2, severe acute respiratory syndrome coronavirus 2, S, spike, VLPs, virus-like particles, vRNP, viral ribonucleoprotein

## Abstract

The severe acute respiratory syndrome coronavirus 2 (SARS-CoV-2), a β-coronavirus, is the causative agent of the COVID-19 pandemic. Like for other coronaviruses, its particles are composed of four structural proteins: spike (S), envelope (E), membrane (M), and nucleoprotein (N) proteins. The involvement of each of these proteins and their interactions are critical for assembly and production of β-coronavirus particles. Here, we sought to characterize the interplay of SARS-CoV-2 structural proteins during the viral assembly process. By combining biochemical and imaging assays in infected versus transfected cells, we show that E and M regulate intracellular trafficking of S as well as its intracellular processing. Indeed, the imaging data reveal that S is relocalized at endoplasmic reticulum (ER)–Golgi intermediate compartment (ERGIC) or Golgi compartments upon coexpression of E or M, as observed in SARS-CoV-2-infected cells, which prevents syncytia formation. We show that a C-terminal retrieval motif in the cytoplasmic tail of S is required for its M-mediated retention in the ERGIC, whereas E induces S retention by modulating the cell secretory pathway. We also highlight that E and M induce a specific maturation of N-glycosylation of S, independently of the regulation of its localization, with a profile that is observed both in infected cells and in purified viral particles. Finally, we show that E, M, and N are required for optimal production of virus-like-particles. Altogether, these results highlight how E and M proteins may influence the properties of S proteins and promote the assembly of SARS-CoV-2 viral particles.

At the end of 2019, SARS-Cov-2 emerged in China through zoonotic transmission and led to the COVID-19 pandemic, cumulating by end of September 2020 to over 31 million cases and more than 950,000 deaths worldwide (1). SARS-CoV-2 belongs to the β-coronavirus genus of the Coronaviridae family that includes SARS-CoV and Middle East Respiratory Virus (MERS-CoV), which are also responsible for severe lower respiratory infections.

The main structural components of coronaviruses are the S (Spike) glycoprotein, the M (Membrane) and E (Envelope) transmembrane proteins, and the N nucleoprotein, which form a viral ribonucleoprotein (vRNPs) complex with the 30kb-long viral genomic RNA (vRNA). The S glycoprotein is the major determinant of viral entry in target cells. The M glycoprotein is key for assembly of viral particles by interacting with all other structural proteins (2, 3), whereas the E protein is a multifunctional protein, supposed to act on viral assembly, release of virions, and pathogenesis (reviewed in (4)). Specifically, through its oligomerization, E forms an ion-channel termed “viroporin” (5, 6). Even though M coordinates virion assembly, an interaction between M and E seems required for the formation of viral particles (7, 8, 9).

Coronaviruses assembly and budding occur in the lumen of the endoplasmic reticulum (ER)–Golgi intermediate compartment (ERGIC) (10, 11). To ensure their accumulation in the ERGIC, M, E, and S proteins contain intracellular trafficking signals that have been identified for some coronavirus species. For example, a dibasic retrieval signal, KxHxx, found at the C-terminus of the cytoplasmic tail of SARS-CoV Spike, allows its recycling *via* binding to COPI (12). Such a recycling of S may increase its chance to interact with M, which resides at the ERGIC, hence inducing S accumulation at the virion budding site.

Here, we aimed at better characterizing the interplay between S and the other structural proteins, *i.e.*, E, M, and N of SARS-CoV-2. Owing to its homology with β-coronaviruses, we hypothesized that some assembly mechanisms might be conserved between SARS-CoV-2 and other β-coronaviruses. Specifically, we aimed at determining how E, M, and N might regulate S intracellular trafficking and maturation, such as its processing by proteolysis, which is not detected for SARS-CoV (13). Furthermore, since SARS-CoV has been proposed to induce the release of S-containing virus-like particles (VLPs) (14), we also aimed at clarifying the minimal set of SARS-CoV-2 proteins required for production of S-containing VLPs.

## Results

### Processing of SARS-CoV-2 spike protein is influenced by other viral proteins

**Figure 1:**
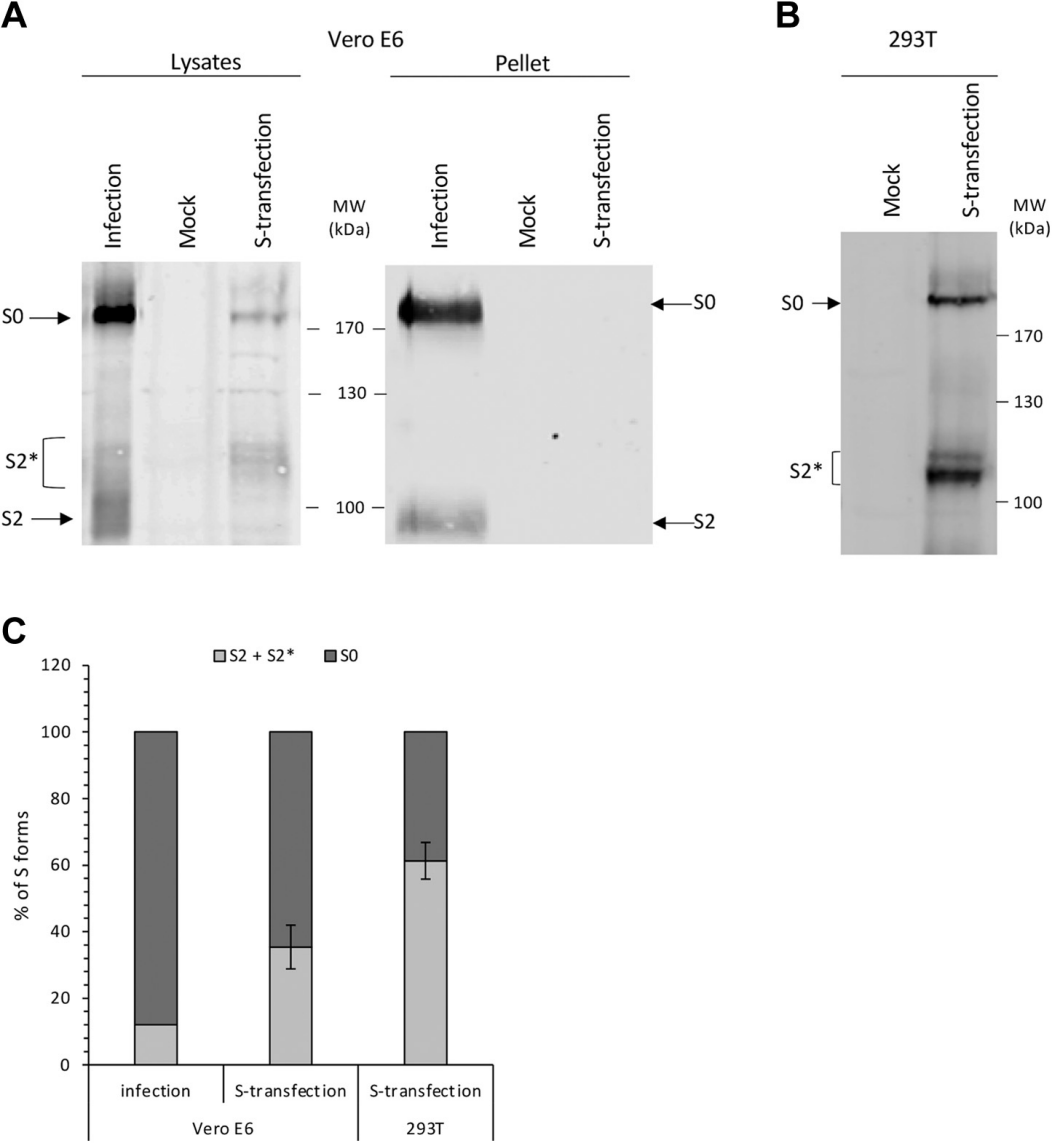
**Processing of SARS-CoV-2 spike protein is influenced by other viral proteins.***A*, representative western blot analysis of cell lysates and pellets of ultracentrifugated supernatants from Vero E6 cells infected by SARS-CoV-2 (infection) or transfected with a plasmid encoding S (S-transfection). *B*, representative western blot analysis of cell lysates of 293T cells transfected with the same plasmid. The blots were revealed using an anti-S2 antibody. The arrows and bracket represent S0, S2, and S2^∗^ forms. *C*, quantification of the proportions of S0 and (S2+S2^∗^) forms in lysates of SARS-CoV-2-infected versus S-transfected Vero E6 or 293T cells as described in (*A* and *B*).

We compared the expression and secretion of the S glycoprotein in Vero E6 cells upon infection with full-length SARS-CoV-2 versus transfection of an S-expressing plasmid at 48 h posttransfection or infection (Fig. 1*A*). We detected in SARS-CoV-2-infected cells and their supernatant both a predominant noncleaved S form, denoted as S0 (of *ca*. 180 kDa), and a cleaved form of S, denoted as S2 (of *ca*. 100 kDa), which is likely induced from S0 processing by furin (13), an ubiquitous protein convertase localized within the cell secretory pathway (15). Interestingly, in S-transfected VeroE6 cells, we observed a lower mobility form of S2 (around 110 kDa), appearing as a doublet band denoted here as S2^∗^, which was predominant as compared with S2 (Fig. 1*A*). In contrast, this S2^∗^ species was poorly detected in SARS-CoV-2-infected cells, which suggested that some other viral proteins influence the maturation of S2. We also detected the S2^∗^ form that was predominantly expressed in 293T cells transfected with S (Fig. 1*B*). Moreover, while both S0 and S2 were the prominent forms detected in purified SARS-CoV-2 viral particles, we did not observe secretion of particles containing S in the supernatant of cells expressing S alone (Fig. 1*A*), suggesting that some other viral proteins are required for secretion of S-containing particles.

Finally, through quantitative western-blot assays, relative to the total of S forms, we detected *ca*. 10% of S2+S2^∗^ in infected Vero E6 cells versus up to 40% in S-transfected Vero E6 cells (Fig. 1*C*), which further indicated that some other SARS-CoV-2 proteins could regulate S processing as well as maturation. We also showed that even stronger S cleavage rate of up to 60% could be detected in transfected 293T cells (Fig. 1, *B*–*C*), indicating that both viral and cellular factors could modulate S0 processing.

### SARS-CoV-2 E and M proteins alter processing and maturation of the S glycoprotein

**Figure 2:**
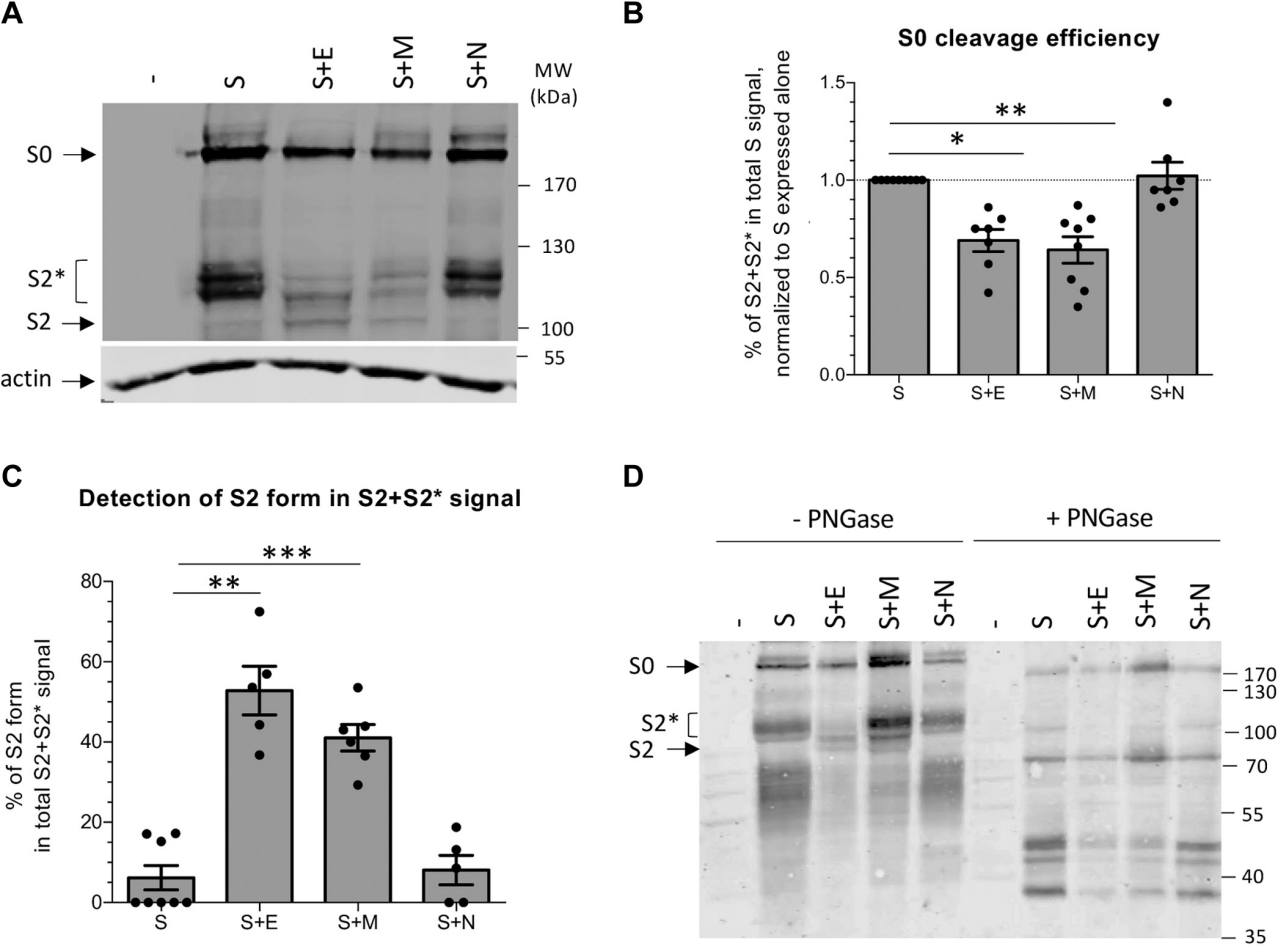
**Coexpression of SARS-CoV-2 E and M alters S processing and maturation.***A*, representative western blot analysis of lysates 293T cells transfected with a plasmid encoding S alone versus S combined with plasmids expressing E, M, or N, as indicated. The blots were revealed using anti-S2 and antiactin antibodies. The arrows and bracket represent S0, S2, and S2∗ forms. *B*, quantification of the percentage of (S2+S2^∗^) forms in the total S signal (S2+S2^∗^+S0) by quantitative western blot analysis as described in (*A*) and normalized to condition when S expressed alone. *C* quantification of the percentage of S2 form in the total S2+S2^∗^ signal by quantitative western blot analysis as described in (*A*). *D*, western blot analysis of cell lysates of 293T cells transfected with a plasmid encoding S alone or S combined with plasmids expressing E, M, or N that were left untreated (–PNGase) or that were treated with PNGase (+PNGase) to remove glycans. The blots were revealed using an anti-S2 antibody. The *arrows* and bracket represent S0, S2, and S2^∗^ forms. The dots on the graphs represent results of independent experiments.

To determine which SARS-CoV-2 proteins could influence S processing and maturation, we coexpressed S with E, M, or N structural proteins in transfected cells. When we determined the ratio of S0/(S2+S2^∗^), we found a strong reduction of S0 cleavage upon S coexpression with E or M (Fig. 2, *A*–*B*). In contrast, coexpression with N did not influence the processing of S, as compared with S expressed alone (Fig. 2, *A* and *B*). Note that the S2 form that was readily detected in SARS-CoV-2-infected cells (Fig. 1*A*) was produced at increased levels upon coexpression of S with E or with M (Fig. 2, *A* and *C*), as compared with S expressed alone or coexpressed with N that mainly yielded the S2^∗^ form. Altogether, these results indicated that both E and M influence processing and maturation of SARS-CoV-2 S.

Since the S protein is highly glycosylated (16), we thought that the variations of S2 versus S2^∗^ forms could reflect differences in their N-glycan maturation profile. To address this possibility, we treated lysates of S transfected cells versus S and E, M, or N cotransfected cells with PNGase F, which removes the N-linked oligosaccharides from glycoproteins. After PNGase F treatment, the profile of these bands was identical whether S was expressed alone versus coexpressed with E, M, or N (Fig. 2*D*). This indicated that S2^∗^ is a glycosylation variant of S2 and suggested that the presence of E or M alters the maturation of S. Note that we also detected smaller forms of S2, between 55 and 70 kDa (Fig. 2*D*), which may represent a cleaved form of S2 or a differential matured form of S2, as previously reported (17).

### Intracellular retention of SARS-CoV-2 S is induced by E and M and prevents cell–cell fusion

Since the E and M proteins of some other coronaviruses are involved in the regulation of S localization (14) and since furin is predominantly found in the late compartments of the cell secretory pathway (15), we reasoned that the difference in SARS-CoV-2 S cleavage rates between infected versus transfected cells (Fig. 1, *A*–*B*) could be due to a difference in S intracellular localization.

**Figure 3:**
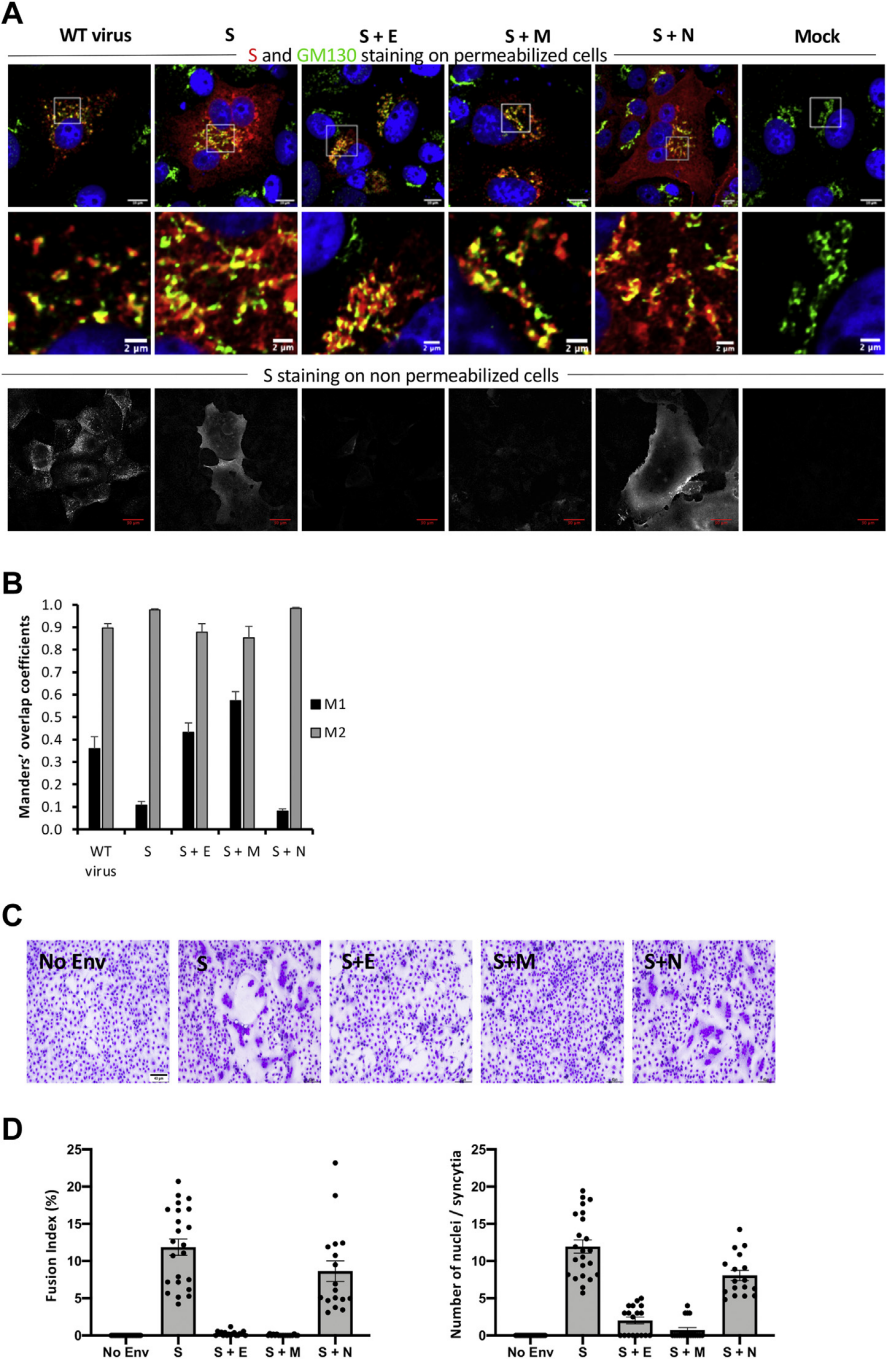
**Expression of SARS-CoV-2 E and M induced the retention of S, which prevents syncytia formation.***A*, representative confocal microscopy images of Vero E6 cells infected or transfected with a plasmid encoding S alone or S combined with plasmids expressing E, M, or N. After cell permeabilization, the cis-Golgi was revealed with the anti-GM130 antibody (*green channel*), the S protein was revealed with the anti-SARS-CoV-2 S1 antibody (*red channel*), and the nucleus was revealed with Hoechst (*blue channel*). Scale bars of panels and zooms from squared area represent 10 μm and 2 μm, respectively (top). The S protein was also revealed on nonpermeabilized cells (bottom). *B*, the Manders’ coefficient M1 represents the fraction of S overlapping with GM130, and the M2 coefficients represent the fraction of GM130 overlapping with S. *C*, representative pictures of syncytia detected in Vero E6 cells transfected with a plasmid encoding S alone or S combined with plasmids expressing E, M, or N. The scale bar represents 40 μm. *D*, fusion index (left) and number of nuclei per syncytia (right) of the different conditions as described in (*C*). The dots on the graphs represent results of independent experiments.

We therefore investigated the cellular localization of SARS-CoV-2 S expressed alone versus coexpressed with other structural viral proteins, as compared with full-length virus. First, we found that the intracellular S detected in SARS-CoV-2-infected Vero E6 cells was predominantly localized in regions that contain GM130, a marker of the *cis*-Golgi but also of compartments close to the ERGIC (18), whereas S expressed alone in Vero E6 cells was widely distributed within the cell (Fig. 3, *A*–*B*). Second, in cotransfected VeroE6 cells, we found that SARS-CoV-2 E or M proteins—though not N—coexpressed with S induced its predominant localization in ERGIC or cis-Golgi as judged by its increased colocalization with GM130 (Fig. 3, *A*–*B*), suggesting that both E and M can modulate the localization of S. Third, through staining of transfected cells without permeabilization, S was not detected at the cell surface when it was cotransfected with E or M (Fig. 3*A*, lower images). Altogether, this indicated that E and M induce the retention of S in GM130-positive compartments.

We found that expression of SARS-CoV-2 S alone induced the formation of syncytia in transfected VeroE6 cells, resulting in the formation in multinucleated giant cells (Fig. 3*C*). This confirmed the presence of S at the cell surface and indicated that all factors required to mediate cell–cell fusion events were present at the surface of these cells. In contrast, we detected strongly reduced number and much smaller syncytia when S was coexpressed with E or M, whereas cell–cell fusion activity induced by S was not significantly changed upon N expression (Fig. 3, *C*–*D*).

Altogether, these results indicated that E and M regulate the localization of S, by allowing its intracellular retention probably within assembly sites in the ERGIC or *cis*-Golgi, which prevents the formation of syncytia by cell–cell fusion. Since the ERGIC and *cis*-Golgi are compartments of the secretory pathway that are located upstream of organelles in which furin is mainly localized (15), this agreed with the poorer processing and maturation of SARS-CoV-2 S upon its coexpression with E and M (Fig. 2).

### SARS-CoV-2 E induces retention of S *via* slowing down the secretory pathway

As shown in Figure 3, coexpression of SARS-CoV-2 S with E induced S intracellular retention. As E of some other coronaviruses is supposed to act as a viroporin (4) and as viroporins of some other coronaviruses (19) or unrelated viruses (20, 21, 22) have been shown to alter intracellular organelles, we hypothesized that SARS-CoV-2 E could induce the retention of S by slowing down the cell secretory pathway. To address this possibility, we wondered if E could impact the secretion of VSV-G tsO45 (VSV-Gts), a temperature-dependent folding mutant of VSV-G, which represents a heterologous viral glycoprotein commonly used as model cargo of protein secretion. At 40 °C, this protein remains unfolded, resulting in its accumulation in the ER, whereas its folding can be restored at 32 °C, which allows its transfer from the ER to the Golgi and then to the plasma membrane.

**Figure 4:**
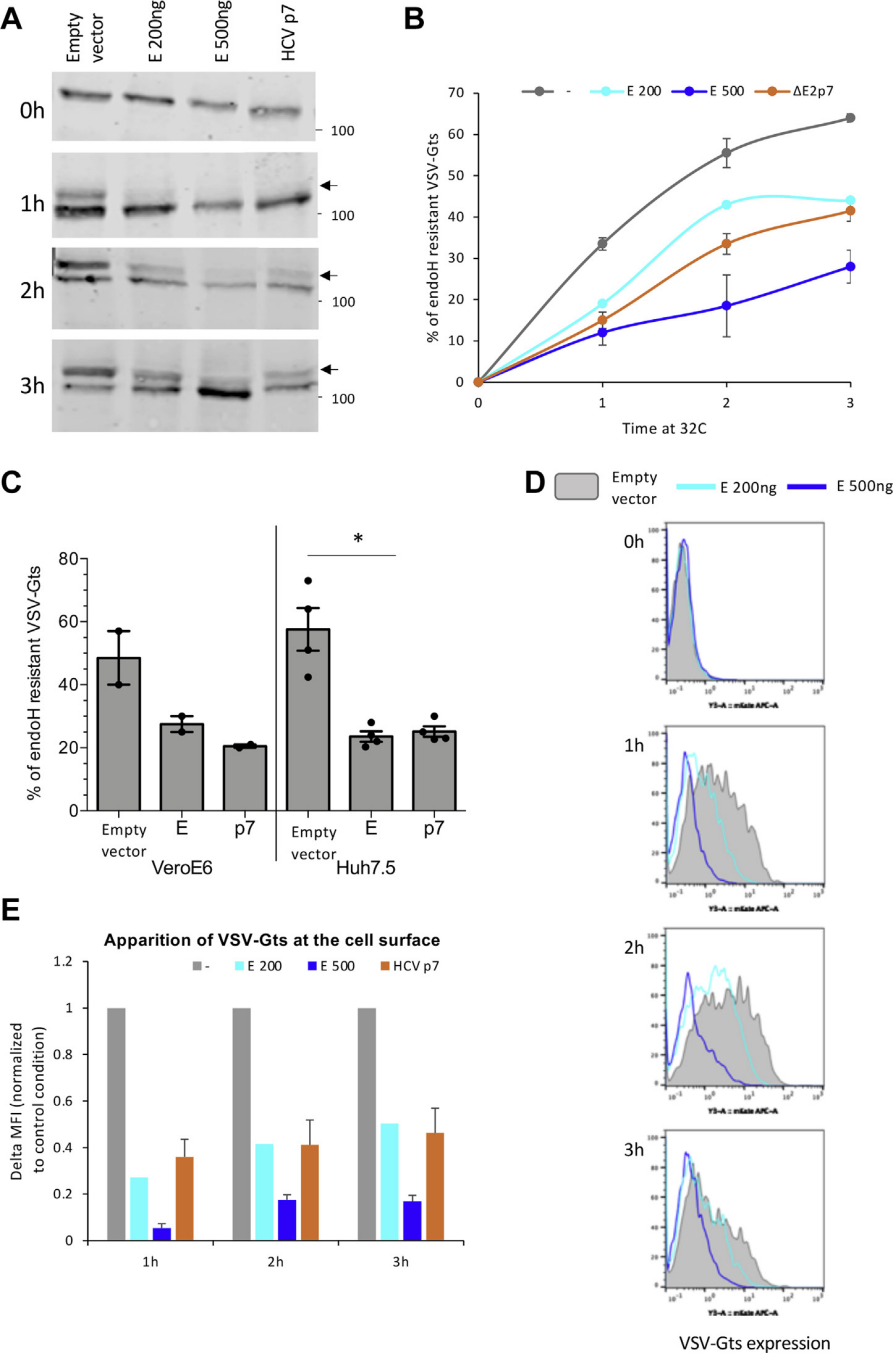
**SARS-CoV-2 E induces the retention of S *via* slowing down the cell secretory pathway.** Huh7.5 or Vero E6 cells were transfected with plasmids encoding a GFP-VSV-Gts fusion protein (referred to as VSV-Gts in the figure and below) and HCV p7 (JFH1) or SARS-CoV E at two different doses, as indicated. Transfected cells were grown overnight at 40 °C, which maintains VSV-Gts unfolded and results in its accumulation in the ER. Cells were then incubated for different periods of time (0 h, 1 h, 2 h, and/or 3h, as indicated) at 32 °C, which allows restoration of its folding and thus, its secretion. *A* representative western blot analysis of cell lysates coexpressing VSV-Gts and E or p7, digested with endoH glycosidase. The blots were revealed using an anti-GFP antibody, allowing the detection of the GFP-VSV-Gts fusion protein. The endoH-resistant VSV-Gts species (*arrows*) indicate proteins that traffic to and beyond the Golgi apparatus. The molecular weight markers are indicated in kDa. *B*, quantification of western blots as described in (*A*). *C*, quantification of western blot analysis of cell lysates of Huh7.5 or Vero E6 cells coexpressing VSV-Gts and E or p7, lysed at 3 h (VeroE6 cells) or 2 h (Huh7.5) posttemperature shifting and digested with endoH glycosidase. The timing was chosen to have the same percentage of endoH resistant forms of VSV-Gts in both cell types. The dots on the graphs represent results of independent experiments. *D,* representative histograms of cell surface expression of VSV-Gts assessed by flow cytometry, using the 41A1 mAb directed against VSV-G ectodomain. *E*, cell surface expression of VSV-Gts as assessed by the variations of the mean fluorescence intensity (delta MFI) of cell surface-expressed VSV-Gts relative to time 0 h at 32 °C. The results were normalized to the control condition (–), in which VSV-Gts was expressed without E or p7.

We transfected Huh-7.5 cells with VSV-Gts in the presence of E or of hepatitis C virus (HCV) p7 used as a positive control (20). First, to address if E alters the traffic from the ER to the *cis*-Golgi, we analyzed the resistance of intracellular VSV-Gts to endoH digestion (23). While at 0h, all VSV-Gts glycans remained endoH-sensitive, reflecting ER retention at 40 °C, they progressively became resistant to endoH cleavage upon incubation at 32 °C for 1–3 h (Fig. 4, *A*–*B*), underscoring VSV-Gts transfer to the Golgi apparatus. We noticed that E expression induced a dose-dependent decrease of the kinetics of VSV-Gts endoH-resistance acquisition (Fig. 4, *A*–*B*). We confirmed these results in transfected Vero E6 cells (Fig. 4*C*). Interestingly, these latter cells have a lower trafficking speed compared with Huh-7.5 cells (compare control conditions in Fig. 4*C*); yet, E was able to slow down the cell secretory pathway in both cell types.

Next, to address the influence of E on trafficking to the plasma membrane, we analyzed the accumulation of VSV-Gts at the cell surface after incubation of transfected cells at 32 °C for different times (Fig. 4*D*). As monitored by flow cytometry analysis, E expression significantly reduced the kinetics and levels of VSV-Gts cell surface expression (Fig. 4, *D*–*E*).

Altogether, these results indicated that SARS-CoV-2 E protein slows down the cell secretory pathway, hence inducing the retention of glycoproteins, which also includes S.

### The C-terminal moiety of SARS-CoV-2 S cytoplasmic tail is essential for M-mediated retention of S

**Figure 5:**
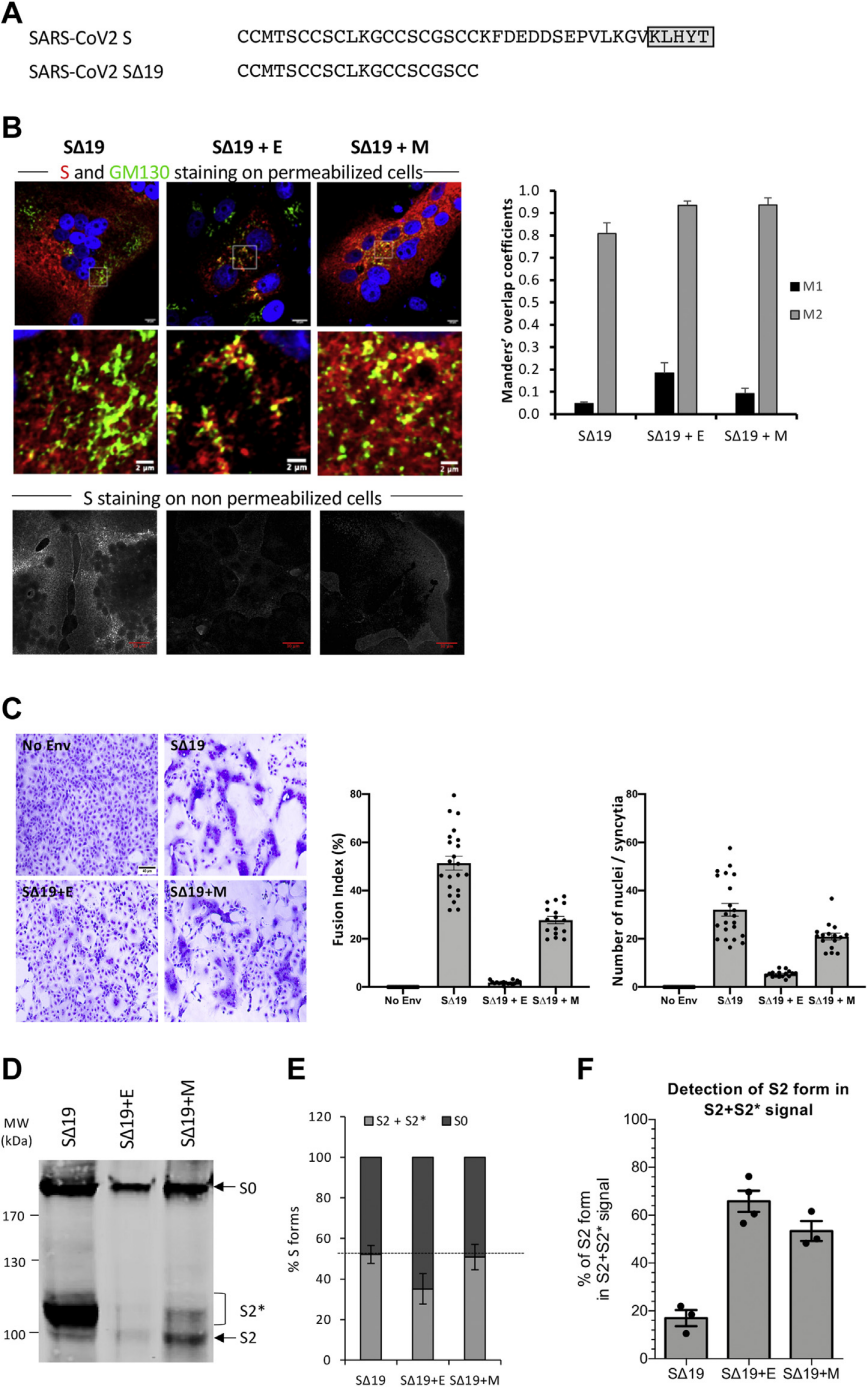
**The C-terminal moiety of S cytoplasmic tail is essential for M-mediated retention of SARS-CoV-2 S.***A*, alignment of sequences of the last amino acids of S of SARS-CoV-2 or mutated by deletion of the last 19 amino acids (SΔ19). The box represents the dibasic retrieval signal. *B*, representative confocal microscopy images of Vero E6 cells transfected with a plasmid encoding SΔ19 alone or SΔ19 combined with plasmids expressing E or M. The cis-Golgi was revealed with the anti-GM130 antibody (*green channel*), the S protein was revealed with the anti-SARS-CoV2 S1 antibody (*red channel*), and the nucleus was revealed with Hoechst (*blue channel*). The Manders’ coefficient M1 represents the fraction of S overlapping with GM130, and the M2 coefficients represent the fraction of GM130 overlapping with S. Scale bars of panels and zooms from squared area represent 10 μm and 2 μm, respectively. The S protein was also revealed on nonpermeabilized cells (*bottom*). *C*, representative pictures of syncytia detected in Vero E6 cells transfected with a plasmid encoding SΔ19 alone or SΔ19 combined with plasmids expressing E or M (*left*). Fusion index and number of nuclei per syncytia determined for the different conditions (*right*). The scale bar represents 40 μm. *D*, representative western blot analysis of 293T cells transfected with a plasmid encoding SΔ19 or SΔ19 combined with plasmids encoding E or M. The blots were revealed using an anti-S2 antibody. The arrows and bracket represent S0, S2, and S2^∗^ forms. *E*, quantification of indicated S forms from independent western blot as described in (*D*). *F*, Quantification of the percentage of S2 forms in the total (S2+S2^∗^) signal by quantitative western blot analysis as described in (*D*). The dots on the graphs represent results of independent experiments.

Previous studies showed that for SARS-CoV S protein, a dibasic retrieval signal KxHxx presents at the C-terminus of its cytoplasmic tail allows S recycling *via* binding to COPI (12). Such a recycling of S increases its capacity to interact with M, which resides at the virion assembly site. Owing to the conservation of this motif in the cytoplasmic tail of SARS-CoV-2 S (Fig. 5*A*), we sought to investigate if the involved mechanism is conserved. Therefore, we tested the impact of M on retention of a mutant of SARS-CoV-2 S, named SΔ19, from which the last 19 amino acids, including the dibasic retrieval signal, were removed (Fig. 5*A*). As compared with wt S, we found that SΔ19, when coexpressed with M in VeroE6 cells, exhibited impaired intracellular retention (compare the Manders’ overlap coefficients (M1) in Figure 5*B* (M1 = 0.1) with those of Fig. 3*B* (M1=0.6)), which confirmed that this retrieval signal allows S recycling and, consequently, M-mediated retention of SARS-CoV-2 S. Moreover, in contrast to the inability of M to induce SΔ19 retention, E coexpressed with SΔ19 could still induce its intracellular retention (Fig. 5*B*), which agreed with our above results that E can induce the retention of S by modulating the cell secretory pathway (Figs. 3 and 4) rather than by directly interacting with S. Of note, we observed that despite the presence of E, SΔ19 did not colocalize with GM130 to the same extent than for S (compare M1 coefficients in Fig. 5*B* (M1 = 0.2) versus Fig. 3, *B* (M1=0.45)). This suggested that E induces the retention of SΔ19 inside the cells although SΔ19 does not accumulate in GM130-containing compartments, likely due to the loss of the retrieval signal.

To corroborate these results, we determined the fusion index of cells expressing SΔ19 alone or SΔ19 in combination with the other viral structural proteins. Interestingly, we found that SΔ19 was highly fusogenic and induced much larger syncytia than wt S (Fig. 5*C* vs. Fig. 3*D*), likely because of its accumulation at the cell surface owing to deletion of the recycling signal. In agreement with our observations that M, but not E, does not alter SΔ19 intracellular trafficking (Fig. 5*B*), the coexpression of SΔ19 and M resulted in formation of syncytia, whereas coexpression of SΔ19 and E almost suppressed cell–cell fusion (Fig. 5*C*).

Next, to confirm the correlation between the intracellular retention of SARS-CoV-2 S and its processing as S2+S2^∗^, we determined the cleavage rate of SΔ19 in cells cotransfected with either E or M. As compared with SΔ19 expressed alone, SΔ19 coexpressed with E exhibited reduced cleavage rate, whereas coexpression of M did not alter its processing (Fig. 5, *D*–*E*). This confirmed that the M-mediated retention of wt S and its reduced cleavage rate are dependent on the C-terminal retention motif, whereas E-mediated retention of wt S and its reduced processing are linked to modification of the cell secretory pathway. Interestingly, we detected increased levels of S2, relative to S2^∗^, when SΔ19 was coexpressed with E or M (Fig. 5, *D* and *F*). Since M is not able to induce retention of SΔ19, this argues for a modification of the N glycosylation pathway by E and M independently of their capacity to induce S retention.

Altogether, these results indicated that E and M induce the retention of SARS-CoV-2 S *via* different mechanisms. Indeed, M induces intracellular retention of S through direct interaction with S, upon its retrieval mediated by its cytoplasmic tail of S, whereas E may induce the retention of S by regulating intracellular trafficking.

### Upon coexpression of other structural proteins, S is incorporated in VLPs independently of its maturation status

Previous reports with alternative coronaviruses indicated that the intracellular retention of S induced by M is essential for assembly of infectious particles and that the presence of E is essential for budding of particles (14). This is in agreement with our above results indicating that M and E induce processing, maturation, and intracellular retention of S, and this suggests that SARS-CoV-2 assembly and budding may share a mechanism common to β-coronaviruses. Thus, we sought to specify the conditions required to induce the formation of SARS-CoV-2 VLPs in our transfection assay.

**Figure 6:**
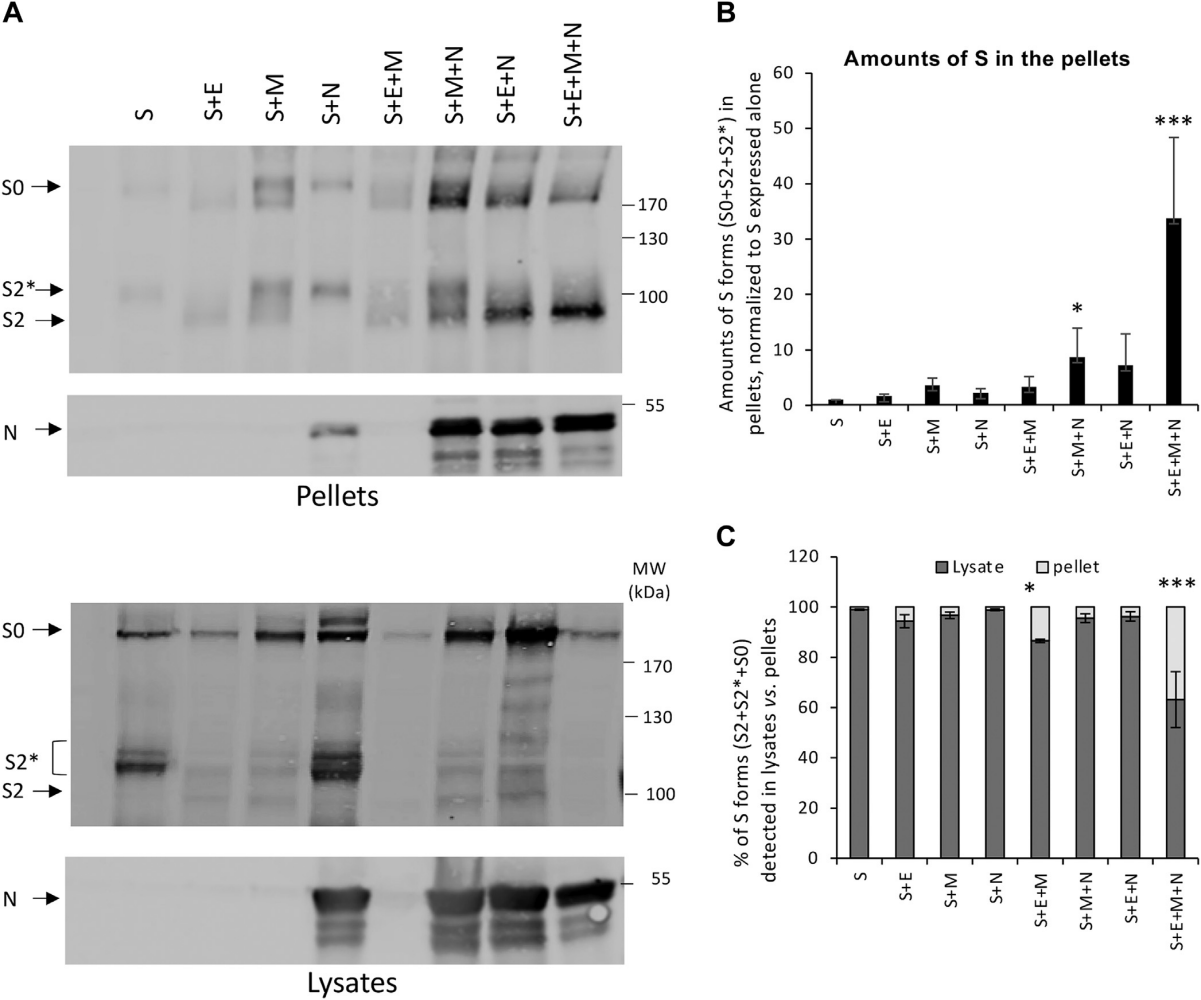
**Secretion of SARS-CoV-2 S-displaying VLPs requires expression of E, M, and N.***A*, representative western blot analysis of cell lysates and pellets from supernatants of 293T transfected with a plasmid encoding S alone or S combined with plasmids encoding E, M, and N. The blots were revealed using an anti-S2 antibody. The arrows and bracket represent S0, S2, and S2∗ forms and N. *B*, the amounts of total S forms (S2+S2∗+S0) detected in pellets of ultracentrifugated supernatants of producer cells were determined by quantification from independent western blots as described in (*A*) and are displayed relative to S expressed alone. *C*, proportion of S forms in lysates and pellets determined by quantification of independent western blot as described in (*A*).

First, we transfected 293T cells with plasmids inducing expression of S alone versus S in combination with E, M, and/or N or with all structural proteins. At 48 h posttransfection, we collected the cell supernatants, and we purified particles by ultracentrifugation through a sucrose cushion. As shown in Fig. 6, *A*–*B*, we found that S expressed alone was poorly detected in the pellets of ultracentrifuged supernatants. Coexpression of E, M, or N with S did not improve the secretion of S. Coexpression of S with both E and N or with both M and N could slightly increase the presence of S in the pellets (Fig. 6, *A*–*B*), though this correlated to an increased expression level in cell lysates (Fig. 6*C*). Remarkably, we found that coexpression of the combination of E, M, and N with S induced a strong production of VLPs with a high-level detection of S in the pellet although, unexpectedly, low level detection in the cell lysate (Fig. 6, *A*–*C*). This indicated that all structural proteins are required for an optimal secretion of S-containing VLPs, which induces their depletion from producer cells. We also found that while N coexpressed with S was poorly secreted, its secretion was readily increased upon coexpression with S, E, and M (Fig. 6*A*), hence suggesting a concerted action of E, M, and N for budding and secretion of SARS-CoV2 S-containing VLPs.

Next, we investigated the VLP incorporation of the S2 versus S2^∗^ forms that were differentially detected in lysates of cotransfected cells (Fig. 2). We found that the S2 form was detected and enriched in the pellets of purified particles produced upon S coexpression with E or M, as compared with those produced with S alone or with S and N (Fig. 6*A*).

Altogether, these results showed that E, M, and N are required for optimal production of VLPs containing S in its N-glycosylation matured forms.

## Discussion

**Figure 7:**
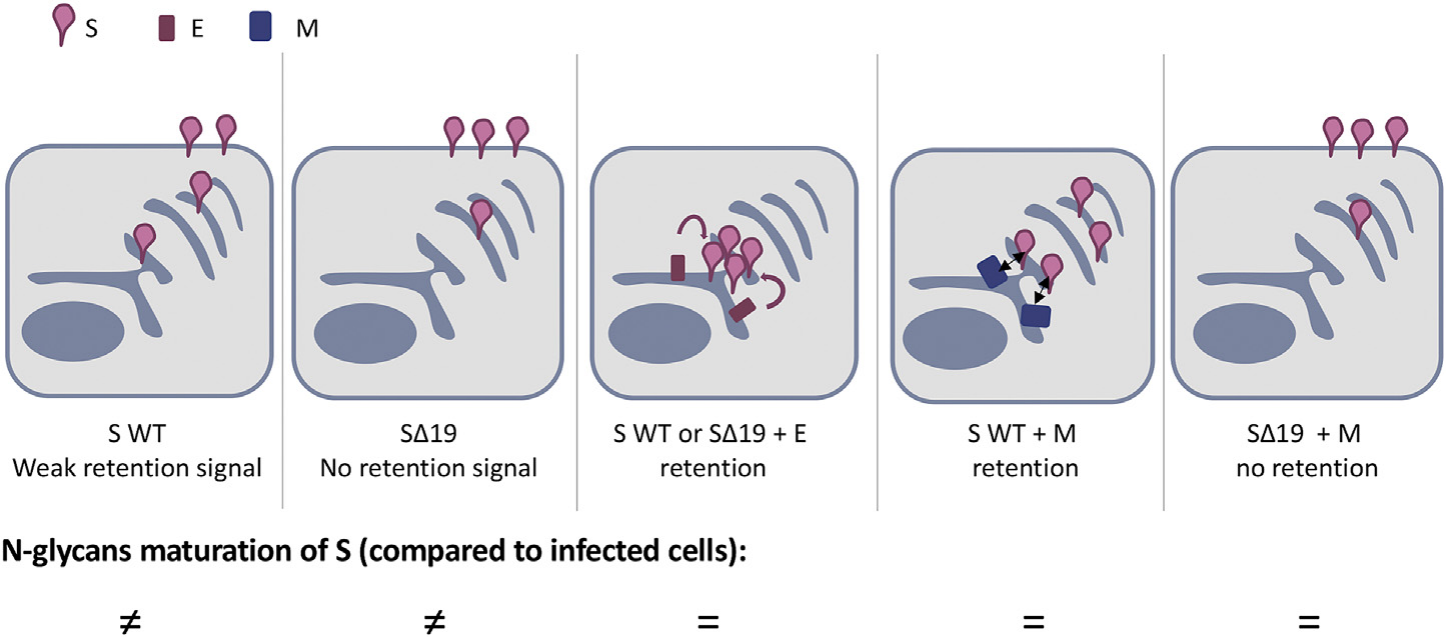
**Model of localization of SARS-CoV2 S protein.** Due to its weak retention signal located at the C-terminus of its cytoplasmic tail, S expressed alone is found at the cell surface but also inside the cells. In contrast, removal of the last 19 amino acids (SΔ19) increases the presence of S at the cell surface. Coexpression of E induces the retention of both wt S and SΔ19 by altering the cell secretory pathway. In contrast, coexpression of M induces the retention of wt S only. Irrespective of S retention signal, the presence of E and M modulates the maturation of N-glycans of S.

Here, we sought to investigate if SARS-CoV-2 shares with other coronaviruses mechanisms of assembly and production of its VLPs. Our results underscore similar mechanisms but also pathways that are unique to SARS-CoV-2 or that had not been highlighted before. Specifically, we found that, by inducing the retention of SARS-CoV-2 S inside the cells, E and M proteins provide a mechanism that not only allows its targeting close to the virion assembly site but also limits its processing to a fusion-active conformation and its cell surface expression, which ultimately prevents syncytia formation. In addition, we also show that independently of their effect on S retention, E or M coexpression with S alters the maturation of the N glycans of S (Fig. 7).

### SARS-CoV-2 E slows down the cell secretory pathway

We show that E induces the retention of S by slowing down the cell secretory pathway (Fig. 4) independently of the retrieval motif harbored by S cytoplasmic tail. Of note, E from alternative β-coronaviruses, and especially SARS-CoV, has been shown to form cation-selective ion channels (24, 25), inferring that SARS-CoV-2 E could be a viroporin, owing to sequence similarity. Interestingly, we and others previously demonstrated that viroporins of unrelated viruses are able to slow down the cell secretory pathway, like for HCV p7 (20) or influenza A virus M2 (21, 22). As it was shown that coronavirus infectious bronchitis virus (IBV) E can alter the secretory pathway (26), we therefore speculate that SARS-CoV-2 E could modify the cell secretory pathway *via* a mechanism shared with some other coronaviruses. Previous reports indicated that the viroporins from divergent viruses can modulate the cell secretory pathway by different mechanisms. For example, the M2 protein from influenza virus has a direct effect on late steps of plasma membrane delivery by delaying late Golgi transport, which indirectly affects the efficiency of earlier transport steps by altering the ionic content of the Golgi apparatus and the endosomes (21, 22). Thus, it is plausible that the modulation of the cell secretory pathway by E could be important for the assembly of infectious particles by allowing the accumulation of the viral structural components at the virion assembly site. Alternatively, the modulation of the cell secretory pathway *per se* could be independent of virion assembly, but rather linked to virulence and/or induction of inflammasome since E was found to be associated to virulence of several coronavirus genera, *e.g.*, for SARS-CoV (27, 28) or IBV (26) as well as induction of the inflammasome for SARS-CoV (29).

### Expression of SARS-CoV-2 E and M modulates the N-glycosylation pathway

We found that E and M regulate the maturation of N glycosylation of S (Fig. 2); yet, we show that this is not related to the role of the former proteins in the retention of S at the Golgi, as shown by using the SΔ19 mutant that retained the same maturation than wt S despite its lack of intracellular retention (Fig. 5). Rather, this suggested that the modification of N-glycosylation is not linked to glycoprotein retrieval in an intracellular compartment lacking the glycosyltransferases. Previous reports have shown that, for other coronaviruses, E and M are located at the ERGIC and/or Golgi membranes (30, 31, 32, 33). Although it was not possible to confirm this for SARS-CoV-2 E and M, owing to the unavailability of specific antibodies, it is likely that they share the ERGIC/Golgi intracellular localization. Since the maturation of N-glycans occurs in the Golgi, one possibility is that accumulation of E and M proteins at the membrane of this organelle could induce changes that alter the correct action of glycosyltransferases (34) and hence, the N-glycan profile of SARS-CoV-2 S. While further studies would be required to determine the role of this modulation of S N-glycosylation maturation, one possibility is that this might modulate virion attachment to some lectins found at the cell surface. A recent study proposed that SARS-CoV S can bind different types of lectin and more particularly LSECtin, which can enhance infection in permissive cells (35). Accordingly, it is possible that, as shown in our report for SARS-CoV-2, the β-coronaviruses have developed mechanisms to control N-glycosylation pathway for their benefit.

### All structural proteins are required for optimal production of SARS-CoV-2 VLPs

While S expressed alone did not induce the secretion of S-containing VLPs, we found that combining its expression with some of the other structural proteins resulted in the formation of VLPs, in agreement with previous results (36), although coexpression of all structural proteins, S, E, M, and N, was the most efficient combination to induce VLP secretion (Fig. 6). Indeed, when all these proteins were expressed, the cells were depleted of S, whereas S was readily detected in the pellets of ultracentrifugated supernatants (Fig. 6*A*). While M is essential for the assembly of virions (14), previous results of others showed that for alternative coronaviruses, S is dispensable for promoting virion assembly although it can be readily incorporated in viral particles upon coexpression with other structural proteins (14). Thus, we propose that SARS-CoV-2 has adopted a similar mechanism for inducing assembly of its particles. For SARS-CoV, the mechanism of formation of VLPs remains unclear since coexpression of M and E (37), of M and N (8), or of M, N and E (38) proteins resulted in the production of VLPs that were not always characterized for their capacity to incorporate S.

Previous results indicated that for most coronaviruses, E and M are essential for the formation of viral particles, implying that a shared mechanism could be used for SARS-CoV-2. First, E and M are known to interact with each other (31). In addition, E might be involved in inducing membrane curvature or scission of vesicles (39, 40). The role of N is more complex and remains poorly defined. N is able to form high-order oligomers (41, 42) even in the absence of RNA (43). In addition, we show that N can be secreted in the presence of S but independently of E and M expression (Fig. 6), suggesting that N may help virion budding when coexpressed with S (44). Indeed, the driving force for budding of enveloped viruses can be provided by the nucleocapsid itself that “pushes” a membranous bud, *via* specific inner structural proteins (*e.g*., Gag precursor of HIV), or alternatively, by the envelope glycoproteins that can form a symmetric lattice “pulling” the membrane (*e.g.*, prME of flaviviruses), even if viruses have evolved and developed different mechanisms with some variations or combinations between these two main models (44). In line with this, we could imagine that for SARS-CoV-2, the optimal driving force for budding could be due to N that could push the membrane as well as to E and M that could create optimal curvature and pull the membrane, hence allowing efficient budding of viral particles that incorporate S.

Altogether, the results of this report indicate that E and M proteins differentially influence the capacity of the S protein to promote assembly of SARS-CoV-2 VLPs. First, E and M are able to induce retention of S in the Golgi/ERGIC compartments. Second, they regulate the N-glycosylation maturation of S. Our results therefore highlight both similarities and dissimilarities in these events, as compared with other β-coronaviruses. Overall, such VLPs could provide attractive tools for studying vaccines or immune responses against COVID-19.

## Experimental procedures

### Cell culture and reagents

Huh7.5 cells (kind gift of C. Rice, Rockefeller University, New York, USA), Vero E6 cells (ATCC CRL-1586), and 293T kidney (ATCC CRL-1573) cells were grown in Dulbecco’s modified minimal essential medium (DMEM, Invitrogen, France) supplemented with 100U/ml of penicillin, 100 μg/ml of streptomycin, and 10% fetal bovine serum.

## Plasmids

*Homo sapiens* codon optimized SARS-CoV-2 S (Wuhan-Hu-1, GenBank: QHD43419.1) was cloned into pVAX1 vector. The delta 19 truncation of S form was generated by site-directed mutagenesis introducing a stop codon after Cys1254 (45). SARS-CoV-2 E, M, and N genes (Wuhan-Hu-1, GenBank: QHD43419.1) were synthesized and cloned into pCDNA3.1(+) vector. The plasmid pEGFP-N3-VSV-Gts was a kind gift from K. Konan, Albany Medical College, USA. The plasmids encoding HCV ΔE2p7(JFH1) were described previously (20).

## Antibodies

Mouse anti-actin (clone AC74, Sigma-Aldrich), rabbit anti-SARS-CoV2 S2, mouse anti-SARS-CoV2 S1 and mouse anti-SARS-CoV2 N (Sino Biological), mouse anti-GFP (Roche), anti-VSV-G (41A1), and rabbit anti-GM130 (clone EP892Y, Abcam) were used according to the providers’ instructions.

### Viral production and infection

SARS-CoV-2 particles (kind gift of B. Lina, CIRI, Lyon) are referenced in GISAID EpiCoVTM database (reference BetaCoV/France/IDF0571/2020, accession ID EPI_ISL_411218) and were amplified on Vero E6 cells (46). Briefly, for stock production, cells were infected with MOI = 0.01 in DMEM for 90 min at 37 °C. Then, medium was replaced with DMEM-2%FCS. Supernatant fluids were collected after 2 days at 37 °C, clarified by centrifugation (400×*g*, 5 min), aliquoted and titrated in plaque forming unit by classic dilution limit assay on the same Vero E6 cells. Lysis and pellet were done as described below.

### VSV-Gts analysis

Huh7.5 cells were seeded 16 h prior to transfection with pEGFP-N3-VSV-Gts and p7- or E-encoding plasmid using GeneJammer transfection reagent (Agilent). Medium was changed 4 h posttransfection and cells were incubated overnight at 40 °C. Twenty-four hours posttransfection, cells were chased at 32 °C. For western blot analysis, cells were lysed at indicated time points in wells cooled on ice before clarification, endoglycosidase Hf treatment, and western blot analysis. Endo-Hf (NEB) treatment was performed according to the manufacturer's recommendations. Briefly, protein samples were mixed to denaturing glycoprotein buffer and heated at 100 °C for 5 min. Subsequently, 1000 units of Endo-Hf were added to samples in a final volume of 25 μl, and the reaction mixtures were incubated for 1 h at 37 °C. For flow cytometry analysis, cells were harvested and put in suspension at 32 °C. At indicated time points, cells were fixed with 3% paraformaldehyde.

### Analysis of expression different proteins in cell lysate and pellet

HEK293 T cells were seeded 24 h prior to transfection with the different plasmids (2 μg of each plasmid for a 10 cm dish) using calcium phosphate precipitation. Vero E6 cells were seeded 24 h prior to transfection with the different plasmids (2 μg of S, 0.2 μg of E, 0.4 μg of M, and 0.8 μg of N corresponding to equimolar ratio of plasmids) using GeneJammer transfection reagent (Agilent). Medium was replaced 16 h posttransfection. Supernatants and cell lysate were done 24 h later. Cell were counted, and 100,000 cells were lysed in 100 μl lysis buffer (20 mM Tris [pH 7.5], 1% Triton X-100, 0.05% sodium dodecyl sulfate, 150 nM NaCl, 5% Na deoxycholate) supplemented with protease/phosphatase inhibitor cocktail (Roche) and clarified from the nuclei by centrifugation at 13,000×*g* for 10 min at 4 °C for quantitative western blot analysis (see below). For purification of particles, supernatants were harvested and filtered through a 0.45 μm filter and centrifuged at 27,000 rpm for 3h at 4 °C with a SW41 rotor and Optima L-90 centrifuge (Beckman). Pellets were resuspended in PBS prior to use for western blot analysis.

### Deglycosylation with PNGase F

PNGase F (NEB) treatment was performed according to the manufacturer’s recommendations. Briefly, protein samples were mixed to denaturing glycoprotein buffer and heated at 100 °C for 5 min. Subsequently, 20 units of PNGase F were added to samples in a final volume of 25 μl with NP-40 and buffer and the reaction mixtures were incubated for 1 h at 37 °C, before western blot analysis.

### Western blot analysis

Proteins obtained in total lysates or after digestion were denatured in Laemmli buffer at 95 °C for 5 min and were separated by sodium dodecyl sulfate polyacrylamide gel electrophoresis, under reducing conditions, then transferred to nitrocellulose membrane, and revealed with specific primary antibodies, followed by the addition of Irdye secondary antibodies (Li-Cor Biosciences). Signals were quantitatively acquired with an Odyssey infrared imaging system CLx (Li-Cor Biosciences).

### Immunofluorescence (IF) and confocal microscopy imaging

Immunofluorescence experiments were done as previously described (47). Briefly, 3 x 10^5^ Vero E6 cells grown on coverslips were infected with wt virus (MOI = 0.01) or transfected with 1 μg of each expressing construct with GeneJammer according to the manufacturer’s instructions. Then, 6 h later, the media of transfected cells was replaced by fresh media and cells were cultured for an additional 18 h. At 24 h postinfection or -transfection, cells were fixed for 15 min with 3% PFA and permeabilized or not with 0.1% Triton X-100. After a saturation step with 3% BSA/PBS, cells were incubated for 1 h with rabbit anti-GM130 and mouse anti-SARS-CoV2 S1 antibodies at 1/200 dilution in 1% BSA/PBS, washed three times with 1%BSA/PBS, and stained for 1 h with donkey anti-rabbit AlexaFluor-488 and donkey antimouse AlexaFluor-555 secondary antibodies (Molecular Probes) diluted 1/2000 in 1% BSA/PBS. Cells were then washed three times with PBS, stained for nuclei with Hoechst (Molecular Probes) for 5 min, washed and mounted in Mowiol (Fluka) before image acquisition with LSM-710 or LSM-800 confocal microscopes.

Images were analyzed with the ImageJ software (imagj.nih.gov) and the Manders’ overlap coefficients were calculated by using the JACoP plugin.

### Cell-cell fusion assay

The cell–cell fusion assay was adapted from (48). Briefly, 3 x 10^5^ Vero E6 cells were transfected with 1 μg of the different expression constructs with GeneJammer according to the manufacturer’s instructions. After 6 h, the transfection media was removed and replaced by fresh media for an additional 24 h. At 30 h posttransfection, transfected cells were fixed and counterstained with May–Grünwald and Giemsa solutions (Sigma-Aldrich) according to the manufacturer’s instructions. Between 17 and 24 fields were acquired in three independent experiments, and the fusion index of the different combinations was determined as (N – S)/T x 100, where N is the number of nuclei in the syncytia, S is the number of syncytia, and T is the total number of nuclei counted.

### Statistical analysis

Significance values were calculated by applying the Kruskal–Wallis test and Dunn’s multiple comparison test using the GraphPad Prism 6 software (GraphPad Software, USA). For fusion index, a two-tailed, unpaired Mann–Whitney test was applied. *p* values under 0.05 were considered statistically significant, and the following denotations were used: ∗∗∗∗, *p* ≤ 0.0001; ∗∗∗, *p* ≤ 0.001; ∗∗, *p* ≤ 0.01; ∗, *p* ≤ 0.05; ns (not significant), *p* > 0.05.

## Data availability

All relevant data are within the article.

## Conflict of interest

The authors declare that they have no conflicts of interest with the contents of this article.
